# A new hat for librarians: providing REDCap support to establish the library as a central data hub

**DOI:** 10.5195/jmla.2018.327

**Published:** 2018-01-02

**Authors:** Kevin Read, Fred Willie Zametkin LaPolla

## Abstract

**Background:**

REDCap, an electronic data capture tool, supports good research data management, but many researchers lack familiarity with the tool. While a REDCap administrator provided technical support and a clinical data management support unit provided study design support, a service gap existed.

**Case Presentation:**

Librarians with REDCap expertise sought to increase and improve usage through outreach, workshops, and consultations. In collaboration with a REDCap administrator and the director of the clinical data management support unit, the role of the library was established in providing REDCap training and consultations. REDCap trainings were offered to the medical center during the library’s quarterly data series, which served as a springboard for offering tailored REDCap support to researchers and research groups.

**Conclusions:**

Providing REDCap support has proved to be an effective way to associate the library with data-related activities in an academic medical center and identify new opportunities for offering data services in the library. By offering REDCap services, the library established strong partnerships with the Information Technology Department, Clinical Data Support Department, and Compliance Office by filling in training gaps, while simultaneously referring users back to these departments when additional expertise was required. These new partnerships continue to grow and serve to position the library as a central data hub in the institution.

## BACKGROUND

REDCap is an electronic data capture system for building and managing online surveys and databases that currently has 2,485 active partners in 114 countries [1]. REDCap is a free, Health Insurance Portability and Accountability Act (HIPAA)–compliant, secure, and low-barrier data collection tool for clinical and translational research that works to ensure data quality and security [2]. The use of data collection tools like REDCap has become essential in biomedical research to keep participant data secure [3] and managed accordingly [4].*

Previously, libraries have used REDCap in collaboration with their research communities to develop a portal of data models [5] and databases for mediating literature searches [6]. Librarians have also begun to provide introductory REDCap training to their communities, with the Pacific Northwest Region of the National Network of Libraries of Medicine offering this service across their region [7].

The authors are positioned in an academic medical center health sciences library’s data services unit and have gained expertise in REDCap through a variety of means. First, we were introduced to REDCap through collaboration with the Emergency Medicine Department to build a REDCap database for morbidity and mortality cases to perform quality improvement. A second and more substantial project involved our participation in a National Library of Medicine informationist project, in which we were tasked with building a REDCap database of more than a 1,000 variables with a complex research workflow for a multicultural dementia screening study [8].

This newly acquired expertise prompted the question of how to integrate our knowledge into existing REDCap support in the medical center. The medical center has one REDCap administrator in the Information Technology (IT) Department who is responsible for maintaining the REDCap system and providing technical support to its users. Additionally, the medical center has a clinical research data management support team called DataCore, which is a fee-for-service unit that provides study design support and ensures that clinical data are standardized and managed accordingly. Both of these groups provide REDCap support in some capacity, but they do not actively provide REDCap training to faculty, students, and staff in the medical center.

The gap in REDCap training across the medical center provided an opportunity for us to establish a partnership with both the REDCap administrator and DataCore to offer REDCap instruction to the medical center community. We held a meeting with both groups about the library’s ability to provide REDCap training across the medical center. The REDCap administrator indicated that the department did not have the capacity to provide wide-scale REDCap training, while DataCore indicated that their department only planned to offer REDCap support in relation to their work with research teams.

Therefore, it was agreed that the library could take on the role of providing REDCap training to the medical center community and would serve as a pivot point for referring detailed study design questions to DataCore and technical questions to the REDCap administrator. Likewise, both the REDCap administrator and DataCore agreed to refer REDCap users to the library should they require introductory REDCap support. The library holds a unique position in the medical center in that we have fostered data-specific relationships with a number of departments through our liaison program [9], making it an ideal platform for promoting REDCap training and support.

## STUDY PURPOSE

This case study describes our effort to offer REDCap services and outlines the service strategy, course offerings, and follow-up consultations that emerged from a collaboration between the health sciences library and our institution’s REDCap administrator and DataCore.

## CASE PRESENTATION

We promoted REDCap training and support using 2 specific methods. First, training sessions were offered through the library’s existing data series, “Data Day to Day,” to gauge the popularity of REDCap training, while simultaneously reaching a large number of people in the medical center. The data series is designed to provide training on data-related topics to anyone in the medical center in the form of 1.5-hour sessions [10]. Second, we promoted the library as a REDCap training provider through posting on the library’s Data Services web page and by marketing the service through the library liaison program. Liaison librarians marketed library REDCap services by reaching out to their communities via email, department newsletters, and faculty meetings. This effort provided an opportunity to establish a targeted REDCap consultation and training service for frontline REDCap support.

### “Data Day to Day” data series

We offered our first REDCap training session as a test case in the summer of 2016. To gauge whether the audience was interested in the topic, the session combined REDCap training with training on the I2B2 tool, which is designed to help integrate and harmonize heterogeneous data from health care and research [11], over the course of the allotted 1.5-hour timeframe. Seventeen people attended this course, and 65% of attendees requested further training in REDCap in their evaluation forms. This outcome resulted in creating a full-length introduction to REDCap course and an advanced REDCap course, which were offered in the fall of 2016 and spring of 2017.

The introductory course focused on highlighting the general functionality of REDCap, including how to create a project, use the online designer to create forms, assign user rights, and export data. The advanced class focused on using the REDCap Shared Library, creating forms using the data dictionary function, importing data from other sources, and using the survey and longitudinal design features. The 2 courses were attended by a total of 70 people who said they will probably (20%) or definitely (79%) use what they learned in their work (1% did not respond), with 100% stating that they would recommend the course they took to others.

**Figure 1:**
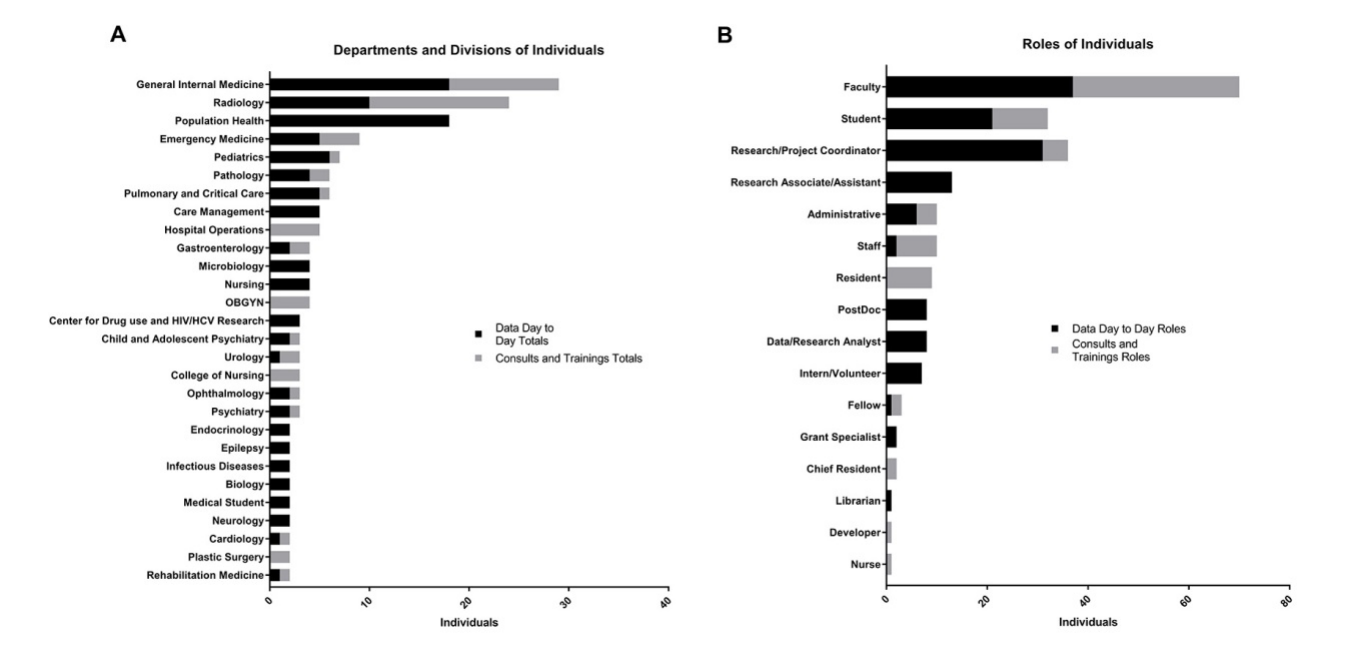
Individual REDCap interactions by department or division and by role

The courses were attended by a diverse audience from different departments and included faculty, students, and staff (Figure 1) from across the medical center, indicating widespread interest in REDCap. Beyond the departments that attended due to targeted liaison outreach (e.g., general internal medicine, radiology), the long tail of different departments that attended provided information about the range of REDCap interest beyond our core groups.

Furthermore, the long tail of interested attendees who were not faculty is also notable in that they highlighted library patrons who were not on our radar for REDCap training. Specifically, the number of project coordinators who attended highlights the potential need for more focused training to this audience, considering their institutional responsibilities in ensuring that staff are trained to use REDCap for data collection and study management. Information about attendees’ roles and departments in the institution also presents an opportunity for the library to reach out to these departments and offer a train-the-trainer approach to these groups, thus making REDCap support more sustainable in the medical center.

Despite the addition of introductory and advanced REDCap courses to the data series, attendees still felt that they would like more advanced REDCap instruction on creating data dictionaries and on designing surveys and longitudinal studies. As a result, we offered three separate courses in the summer of 2017: “Introduction to REDCap”; “Advanced REDCap: Creating Data Dictionaries, Using the REDCap Shared Library & Data Import Tool”; and “Advanced REDCap: Surveys and Longitudinal Studies.” These courses provided us with an opportunity to gauge whether researchers were interested in these topics and whether they would satisfy the need for more advanced training.

These 3 courses were attended by a total of 54 people, who highly rated the courses by indicating in the evaluation forms that they would probably (17%) or definitely (80%) use what they learned in their work (3% did not respond). All (100%) attendees said they would recommend the courses to others. The de-identified data, certified as exempt from the institutional review board, are available as supplementary files.

Several attendees in the advanced data dictionaries class requested advanced training in topics offered in the survey and longitudinal design class and vice versa, indicating that we had developed the appropriate content to address the needs of our community. Finally, in the evaluation form’s free-text question about topics of future interest, all of the suggested topics after the introductory course were addressed in the two advanced courses, further indicating that our approach was effective and our topic coverage was appropriate.

With regard to our collaboration with the REDCap administrator and DataCore, we contacted the REDCap administrator before each data series to notify the administrator of a potential increase in support requests due to our classes. Throughout the data series, the REDCap administrator served as a key resource for responding to questions that we could not answer when the classes were taught. We also referred attendees to the REDCap administrator if they had further questions about any of the technical aspects of REDCap. The library also invited the DataCore director to teach a clinical data management course during the same week that we provided REDCap training to help attendees make connections between data management best practices and use of REDCap for these purposes.

While “Data Day to Day” was an excellent method for teaching REDCap to a broad range of researchers, we wanted to ensure that we could provide support beyond the data series, which was only offered three times a year. Therefore, “Data Day to Day” was used as a springboard to market targeted REDCap consultation and training services to researchers who attended the classes.

### Targeted consultation and training service

**Figure 2:**
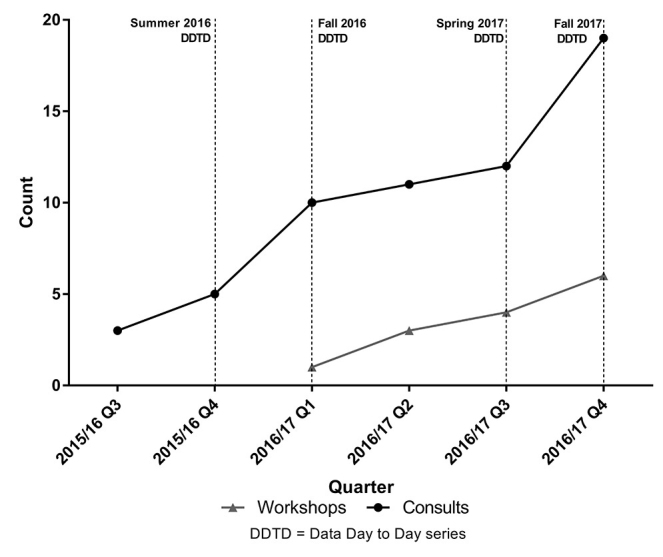
Consultations and workshops over time

To date, we have fielded 75 REDCap training and consultation encounters including phone support (5%), email support (29%), tailored instruction for research groups and residents (19%), and one-on-one consultations with faculty, students, and staff (47%). Using “Data Day to Day” as a way to market the library service has not only been effective in gaining interest from a wide range of departments and roles across the medical center (Figure 1), but has also resulted in an increase in consultation and training opportunities after each “Data Day to Day” series (Figure 2). Consultations comprise phone, email, and in-person interactions, while workshops specifically refer to tailored instruction.

We tracked how the medical center heard about the library offering REDCap services using our usage statistics that are monitored in a REDCap database. The majority of referrals have been through a liaison librarian (31%) or previous data services encounter (29%), with the rest coming from “Data Day to Day” participants (17%), referral from a student or faculty member who recommended our services (7%), our library ticketing system (4%), and users who engaged with library resources such as the library website (4%). Eight percent of referrals were not reported. Considering liaison referrals have been our most effective outreach measure, this suggests that marketing REDCap via the liaison program was an effective approach.

**Figure 3:**
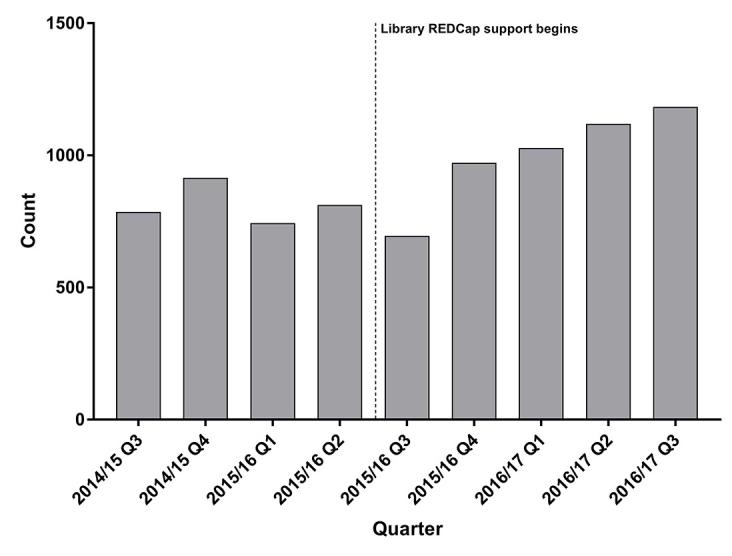
REDCap users by quarter

Regarding referrals to the REDCap administrator and DataCore, the library has referred fourteen users to the REDCap administrator for technical support, eleven users to DataCore for study design assistance, and three users to other liaison librarians for library-related searching projects, while the REDCap administrator has referred users to the library thirty-five times for REDCap training. This collaboration between groups has been effective in increasing support for users. Since the library has begun to offer REDCap training and support, there has also been a steady increase in active REDCap users each quarter (Figure 3).

The REDCap administrator has indicated that each time a “Data Day to Day” series is held, there is a spike in new users. This may, in part, be due to the fact that we ask attendees to sign up for a REDCap account before attending our sessions so that they can participate in class activities. Thus, the growth in REDCap users may reflect both the number of people who use the tool and the high level of attendance at our classes.

To help foster the REDCap community in the medical center, we have also established a REDCap Forum—an internal message board system at the medical center—in collaboration with the REDCap administrator to serve as a platform where REDCap users can ask questions about their projects and share tips that they have discovered when using the system. The forum currently has twenty-three active users, and the library and the REDCap administrator share the duties in responding to questions as needed.

## DISCUSSION

The opportunity for the library to provide a REDCap training and consultation service in the medical center has been beneficial for helping researchers associate the library with data. Furthermore, it has provided us with a venue for identifying additional data-related support needs. The data services unit in the library offers a range of services including training for data management, data visualization, and programming tools (e.g., R, MATLAB), and each REDCap consult or training instance can lead to a referral to these other data services. These interactions are a learning opportunity to gain insights into the types of research being completed in the institution, which serves to inform the data services unit about future service development.

Our partnership with the REDCap administrator and DataCore has also increased communication between the groups and improved communication about REDCap issues more generally, thus creating a strong support network in the institution. The collaboration with the REDCap administrator and DataCore has continued in the form of meetings where we discuss the implementation of the service model and referrals. Each group, respectively, continues to refer REDCap users to the appropriate group for support, and together, we work to address difficult REDCap requests via email and in-person communication. This effort has established the library as a centralized place to provide data support and referrals in collaboration with other medical center groups.

### Challenges

While there are many benefits to offering REDCap services, there are several challenges as well. The persistent challenge has been meeting the demand for training and consultations across the medical center. We have been careful about limiting the promotion of library REDCap services to a few liaison communities for targeted outreach, while keeping the rest of the training isolated to the “Data Day to Day” series to reach a broader audience.

As the popularity of REDCap increases and incoming mandates from the medical center state that research data must be stored in REDCap, the library will require additional resources from the institution to support this effort. For instance, our institution could adopt an approach similar to the TRAIL program at the University of Washington Health Sciences Library, where six librarians offer beginner REDCap training with support from their Clinical and Translational Science Institute and Department of Medicine [7].

Because we offer more than beginner REDCap training and provide tailored hands-on consultations and workshops as part of our service, our model would require additional personnel with significant REDCap expertise. Additionally, with REDCap use growing rapidly, questions must be answered as to the viability of the library becoming a REDCap training shop versus offering a train-the-trainer approach for individual departments to reduce the burden on the library.

To meet the existing demand for training, three librarians (two in our data services unit and one population health librarian) have begun attending classes that we teach and building their own projects in REDCap to train themselves. However, library leadership has been hesitant about training librarians in REDCap while the future sustainability of REDCap support is still being discussed. Until the library has carved out a role for itself in the institution where it receives adequate resources to provide full-scale REDCap support, there will be no additional REDCap training for librarians. However, for librarians looking to gain skills in using REDCap, we have found the most effective method is to practice building projects in the tool, such as the critical experience we gained from participating in the informationist project. Beyond building projects, the REDCap administrator has been an excellent resource for helping us become experts in using REDCap.

### Future directions

As our REDCap service continues to evolve, we plan to take a number of next steps in collaboration with the REDCap administrator and DataCore. First, we plan to create additional courses that focus on REDCap logic for creating calculations and linking data collection instruments, using the randomization module, and practicing data security. Both the randomization module and data security courses would be offered in collaboration with the REDCap administrator and members of the compliance office, who have significant expertise in these areas, thus leading to new partnerships with data-specific departments.

Additionally, we are collaborating with DataCore to develop a series of clinical data management classes in which REDCap will be used as an example of a tool that promotes best practices in clinical data management. This opportunity will bring experts in data standards, Food and Drug Administration (FDA) compliance, and study design from DataCore to teach alongside library staff, which will serve to expand our service offerings and strengthen our library’s position as a centralized data service.

The REDCap administrator, DataCore, and the library have begun discussions about what a sustainable model for offering REDCap services will look like going forward. One component of this model would include a comprehensive, coordinated REDCap service across all departments and institutes, whereby department personnel are trained to provide REDCap training to their faculty, students, and staff and are integrated into curricula or the onboarding process, with the library serving as coordinator in this effort. Another would be the widespread sharing of REDCap information and questions raised by users that could be mined and searched in a centralized location to help facilitate quicker responses to questions and eliminate the burden of having individualized in-person meetings on the same issues.

We view REDCap consultations and training as a service in which libraries can permanently add to their list of long-standing service offerings like literature searching and citation management. With support from the institution and careful planning from the library on the sustainability of the service, REDCap as a service can provide libraries with valuable insights into the types of research being conducted at their institutions and serve to identify researchers’ data management challenges. Offering REDCap services presents opportunities for libraries to develop partnerships and strengthen relationships with medical center departments including IT, compliance offices, and clinical data support. With the establishment of new partnerships and service offerings across an institution, providing REDCap support can help libraries become a hub for data in their institutions.

## Supplemental Files

Appendix A REDCap data set

Appendix B Data collection and analysis instructions

Appendix C Data Day to Day evaluation form

## Footnotes

* Patridge and Bardyn review REDCap in this issue of the *Journal of the Medical Library Association:* DOI: http://dx.doi.org/10.5195/jmla.2018.319.
