# Supplementary material for: A new hat for librarians: providing REDCap support to establish the library as a central data hub
Source: J Med Libr Assoc. 2018 Jan 2;106(1):120–6. doi: 10.5195/jmla.2018.327 (PMC5764577; doi:10.5195/jmla.2018.327)
Supplement: Appendix B [file jmla-106-120-s002.pdf]

Journal of the Medical Library Association

Supplemental content to
J Med Libr Assoc. Jan;106(1):dx.doi.org/10.5195/jmla.2018.327
www.jmla.mlanet.org
© Read, LaPolla 2018

A new hat for librarians: providing REDCap support to establish the library as a central data hub

Kevin Read; Fred Willie Zametkin LaPolla

APPENDIX B

Data collection and analysis instructions

#Data Day to Day Data
- all data were collected on paper in classes using the evaluation form "REDcapPaper_DDTD_Evaluation", and then transferred into a REDCap database with the same questions

#Training and consultation data
- all data were entered into a REDCap database after a training session or consultation was completed

#Master dataset
- all data were exported from REDCap version 7.0.20 into a csv file
- the csv was then transformed into Microsoft Excel, and responses were grouped and analyzed using Excel's COUNTIF function
- data were then visualized using GraphPad Prism version 7.02 using the tool's Grouped Column charts, Column charts, and XY line charts to create the figures within the paper
