# Supplementary material for: A new hat for librarians: providing REDCap support to establish the library as a central data hub
Source: J Med Libr Assoc. 2018 Jan 2;106(1):120–6. doi: 10.5195/jmla.2018.327 (PMC5764577; doi:10.5195/jmla.2018.327)
Supplement: Appendix C [file jmla-106-120-s003.pdf]

JMLA Journal of the Medical Library Association

SC

Supplemental content to
J Med Libr Assoc. Jan;106(1):dx.doi.org/10.5195/jmla.2018.327
www.jmla.mlanet.org
© Read, LaPolla 2018

A new hat for librarians: providing REDCap support to establish the library as a central data hub

Kevin Read; Fred Willie Zametkin LaPolla

APPENDIX C

Data Day to Day evaluation form

Class:
Instructor:

School:
- School of Medicine
- College of Dentistry
- College of Nursing
- Tisch Hospital
- Other: ____________________

Department/Division: ____________________

What is your role (e.g. postdoc, faculty, student, intern, administrator)?

____

What did you hope to get out of this class and to what degree did you get that?

____

Would you recommend this class to others?
- Highly recommend
- Recommend
- Recommend with reservations
- Not recommend

Will you use what you learned in this class for your work?
- Definitely will
- Probably will
- Probably won't
- Definitely won't

Was the level of the material presented?
- Too low
- Just right
- Too advanced

Was the length of time allotted for this topic?
- Too short
- Just right
- Too long

JMLA Journal of the Medical Library Association

SC
Supplemental content to
J Med Libr Assoc. Jan;106(1):dx.doi.org/10.5195/jmla.2018.327
www.jmla.mlanet.org
© Read, LaPolla 2018

Was the material effectively presented?
- Very effectively presented
- Mostly effectively presented
- Somewhat effectively presented
- Not effectively presented

Would you be interested in more advanced topics in the area of this class?
- Yes
- No

If yes, are there any topics in particular you would like to see?

___

What other topics would you be interested in seeing offered in future Data Day to Days?

___

Please share any additional comments about the Data Day to Day series or this class:

___
